# Efficacy of Nerve-Derived Hydrogels to Promote Axon Regeneration Is Influenced by the Method of Tissue Decellularization

**DOI:** 10.3390/ijms23158746

**Published:** 2022-08-06

**Authors:** Vijay Kumar Kuna, Andre Lundgren, Luis Oliveros Anerillas, Peyman Kelk, Maria Brohlin, Mikael Wiberg, Paul J. Kingham, Ludmila N. Novikova, Gustav Andersson, Lev N. Novikov

**Affiliations:** 1 Department of Integrative Medical Biology, Umeå University, SE-901 87 Umeå, Sweden; 2 Department of Surgical and Perioperative Science, Section of Hand and Plastic Surgery, Umeå University, SE-901 87 Umeå, Sweden; 3 Department of Odontology, Umeå University, SE-901 87 Umeå, Sweden; 4 Department of Clinical Microbiology, Umeå University, SE-901 87 Umeå, Sweden; 5 Wallenberg Centre for Molecular Medicine, Umeå University, SE-901 87 Umeå, Sweden

**Keywords:** biosynthetic conduit, decellularized nerve tissue, diffusion tensor imaging, MRI, nerve-derived hydrogel, peripheral nerve injury

## Abstract

Injuries to large peripheral nerves are often associated with tissue defects and require reconstruction using autologous nerve grafts, which have limited availability and result in donor site morbidity. Peripheral nerve-derived hydrogels could potentially supplement or even replace these grafts. In this study, three decellularization protocols based on the ionic detergents sodium dodecyl sulfate (P1) and sodium deoxycholate (P2), or the organic solvent tri-n-butyl phosphate (P3), were used to prepare hydrogels. All protocols resulted in significantly decreased amounts of genomic DNA, but the P2 hydrogel showed the best preservation of extracellular matrix proteins, cytokines, and chemokines, and reduced levels of sulfated glycosaminoglycans. In vitro P1 and P2 hydrogels supported Schwann cell viability, secretion of VEGF, and neurite outgrowth. Surgical repair of a 10 mm-long rat sciatic nerve gap was performed by implantation of tubular polycaprolactone conduits filled with hydrogels followed by analyses using diffusion tensor imaging and immunostaining for neuronal and glial markers. The results demonstrated that the P2 hydrogel considerably increased the number of axons and the distance of regeneration into the distal nerve stump. In summary, the method used to decellularize nerve tissue affects the efficacy of the resulting hydrogels to support regeneration after nerve injury.

## 1. Introduction

Peripheral nerve injuries (PNI) are common and often affect economically active young adults [1]. Estimated annually, the European incidence of PNI affecting the hand is 140 per million inhabitants [2]. The PNI could be caused by mechanical trauma from domestic, occupational, or road traffic accidents, following tumour surgery, or occur as a result of diabetes [1]. The injury often results in lifelong disability due to sensory and motor deficits, and neuropathic pain, which require rehabilitation and therapy [3].

Despite modern microsurgical techniques of PNI repair, functional restoration is often incomplete [1,4]. In patients with lost nerve tissue, where the two nerve stumps cannot be directly reconnected without tension, the resulting ‘‘nerve gap’’ is reconstructed using autologous nerve grafts. However, the outcomes following nerve grafting are relatively poor and worsen with increasing gap length [1,5]. In addition, there is limited donor tissue availability for large peripheral nerve defects and surgery induces further donor site morbidity [6].

A tissue engineering approach could potentially provide an alternative method to overcome the shortage of donor material for reconstruction of injured large peripheral nerves. Bridging nerve conduits for PNI repair have been manufactured from various natural and synthetic materials [6,7]. Tubular collagen conduits (Neuragen, Neuroflex, Neuromaix, and Revolnerv), conduits made from polyglycolic acid (Neurotube) and polycaprolactone (Neurolac), are FDA and CE certified and are used clinically. However, these conduits have been found to be most effective for the repair of relatively small nerve defects [1,6]. Different structural modifications, such as micropatterning or nanopatterning of the tubular conduits [8] and luminal filling with bioactive substances, such as cells and cell-derived products [9], extracellular matrix (ECM) molecules [10,11], growth factors [12,13], and filamentous structures [14], have also been tested in preclinical studies with varying limited additional benefits.

Recently, a promising treatment strategy based on decellularized peripheral nerve tissue has been developed [15]. Decellularized nerve grafts may be allografts or xenografts completely devoid of donor cells, but with the retention of significant amounts of ECM which support cell migration and axon growth following PNI [15]. In addition, they evoke a very limited inflammatory response following transplantation [16,17]. Removal of cells from the donor material is performed using physical, chemical, or enzymatic methods [18]. However, careful selection of reagents and exposure time are required for minimizing the potential damage to the nerve ECM. Several decellularization protocols for peripheral nerves have already been tested [19,20,21], and analysis of the optic nerve ECM has shown that it shares characteristics of embryonic ECM with decreased levels of growth-inhibitory molecules [22].

Human decellularized allografts have been introduced clinically by AxoGen (Alachua, FL, USA) and are shown to be safe and effective in repairing relatively small nerve defects [6,23,24,25]. However, the efficacy of decellularized nerve grafts for reconstruction of large nerve defects remains controversial [6,21,23,24,25,26], which could be attributed to the lack of donor Schwann cells, limited migration of the host cells into the graft, poor axon regrowth through the collapsed endoneurial tubes, and delayed angiogenesis [27,28]. Hydrogels prepared from the decellularized nerve tissue could provide a natural source of nerve-derived ECM. They can be easily combined with existing FDA-approved tubular conduits and can be supplemented with cells, cell products, or growth factors.

In this study, we developed novel protocols for decellularization and hydrogel preparation from porcine vagus nerves, and assessed the efficacy of the hydrogels to support cultured Schwann cells and sensory neurons. Furthermore, using magnetic resonance imaging (MRI) and traditional histological techniques, we investigated the ability of the hydrogels to stimulate axon regeneration after sciatic nerve injury and repair in adult rats.

## 2. Results

### 2.1. Characterisation of Decellularized Nerve Tissue

**Figure 1:**
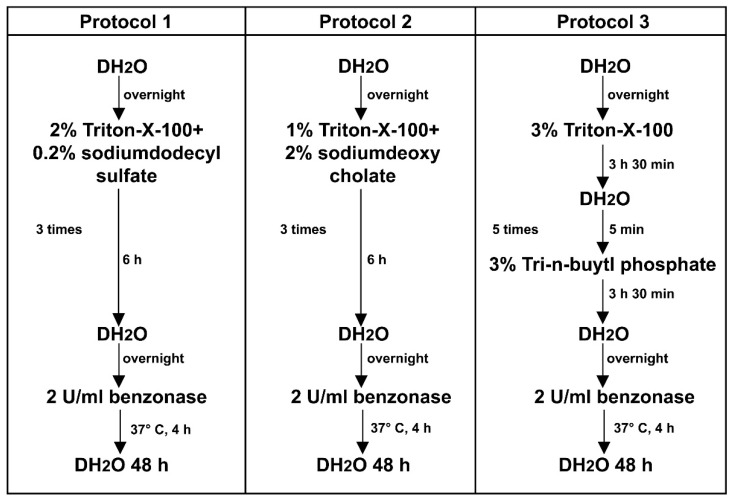
**Decellularization protocols.** Schematic diagram showing different steps of the nerve tissue processing, using protocols P1, P2, and P3.

Porcine vagus nerves were decellularized using three different protocols, based on a combination of non-ionic surfactant and Triton™ X-100 emulsifier with the ionic detergents sodium dodecyl sulfate (SDS) in protocol P1 and sodium deoxycholate (SDC) in protocol P2, or the organic solvent tri-n-butyl phosphate (TnBP) in protocol P3 (Figure 1).

**Figure 2:**
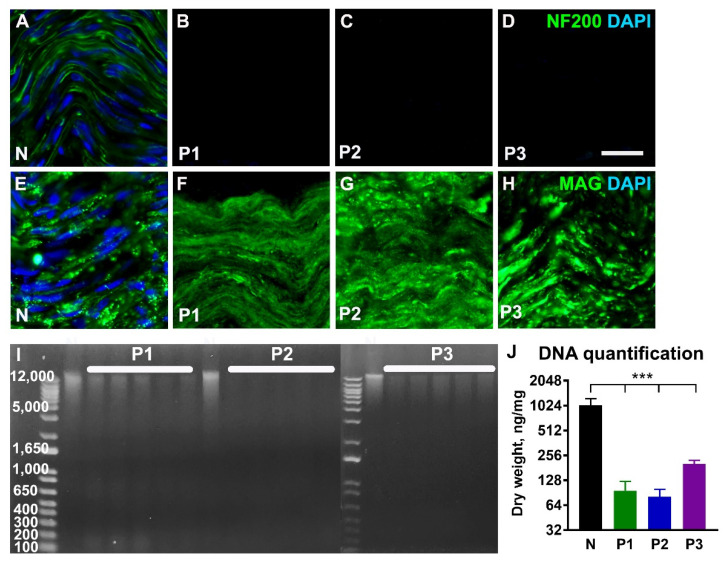
**Characterization of the decellularized nerve tissue.** Representative longitudinal sections from normal nerve (N) and decellularized nerves following treatment with protocols P1, P2, and P3 and immunostained for axonal neurofilaments (NF 200, (**A**–**D**)) and myelin-associated glycoprotein (MAG, (**E**–**H**)). Cell nuclei are counterstained with DAPI. The agarose gel electrophoresis in ((**I**), *n* = 5) and histogram in ((**J**), *n* = 5) shows significant reduction of the genomic DNA following treatments. Error bars show the S.E.M. *p* < 0.001 is indicated by ***. Scale bar, 25 µm.

Immunostaining of the decellularized nerves for neurofilaments in axons (NF 200, Figure 2A–D) demonstrated complete absence of NF 200-positive profiles after using all three tested protocols. Staining for myelin-associated glycoprotein (MAG, Figure 2E–H) was largely preserved after application of the P1 and P2 protocols. However, following treatment with the P3 protocol, we consistently found small areas in the nerves devoid of MAG-labelling. Counterstaining of the nerve tissue sections with DAPI showed the absence of nuclei after treatment with all three protocols (Figure 2B–D,F–H). DNA quantification showed a significant (*p* < 0.001) decrease in the amount of DNA in decellularized nerves when compared with normal untreated nerve tissue (Figure 2I,J). The remaining genomic DNA was around 100 bp in length.

**Figure 3:**
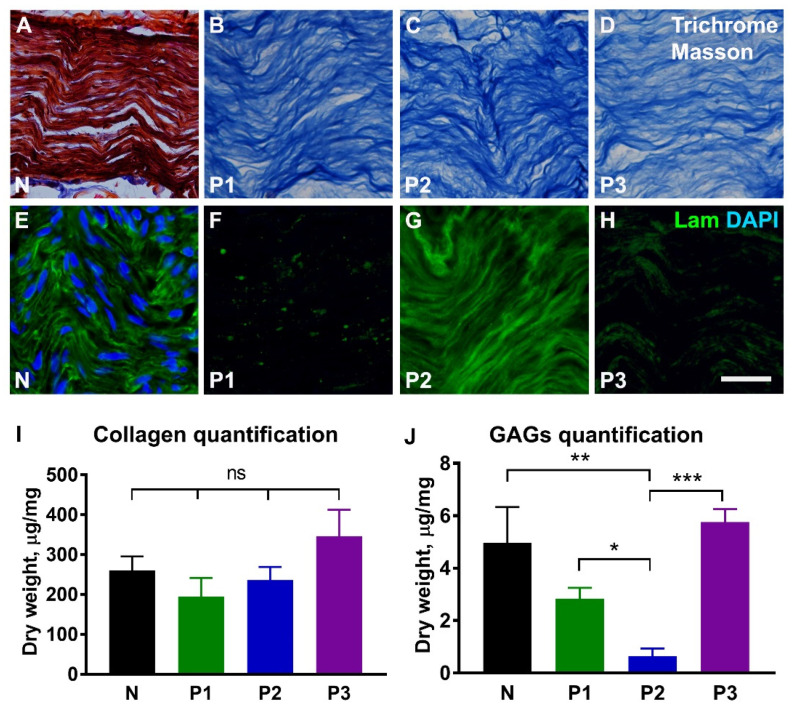
**Extracellular matrix in the decellularized nerve tissue.** Representative longitudinal sections from normal nerve (N) and decellularized nerves following treatment with protocols P1, P2, and P3, and stained for collagen (**A**–**D**) and laminin (**E**–**H**). The red colour in (**A**) corresponds to the cell cytoplasm in normal nerve. Histograms in (**I**,**J**) show quantitative analysis of insoluble fibrillary collagen (*n* = 5) and sulfated glycosaminoglycans (GAGs, *n* = 5). Note strong laminin immunostaining following treatment with P2 protocol. Error bars show the S.E.M. *p*-values are indicated as follows: * *p* ≤ 0.05, ** *p* ≤ 0.01, and *** *p* ≤ 0.001; ns, not significantly different. Scale bar, 25 µm.

Analysis of extracellular matrix proteins showed that collagen was preserved in all nerve samples when compared with untreated nerves (Figure 3A–D,I), whereas laminin was only found in nerves treated with the P2 protocol (Figure 3E–H). Quantification of glycosaminoglycans revealed a significant decrease following treatment with P2 protocol when compared with both the naïve nerve and nerves treated with protocols P1 and P3 (*p* ≤ 0.05; Figure 3J).

### 2.2. Characterisation of Hydrogels Prepared from Decellularized Nerve Tissue

**Figure 4:**
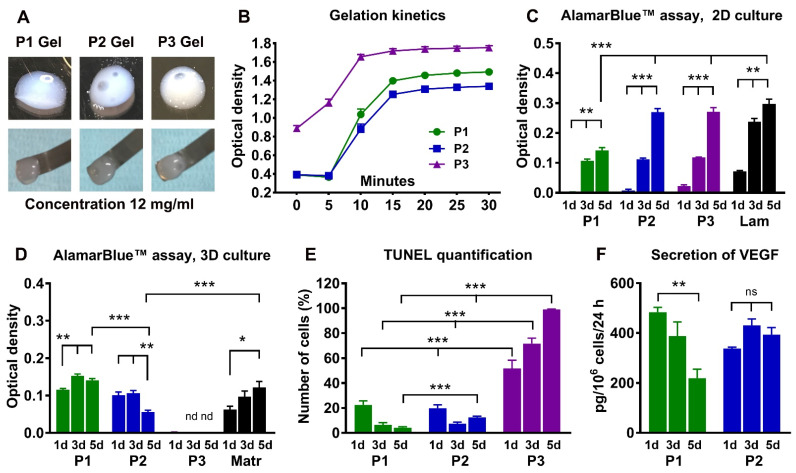
**Characterization of the nerve-derived hydrogels.** Images and histogram in (**A**,**B**) show appearance and handling of the 12 mg/mL P1, P2, and P3 hydrogels after solidification, and their gelation kinetics (*n* = 5). Histograms in (**C**,**D**) demonstrate Schwann cell proliferation and viability at 1, 3, and 5 days in the wells coated with P1, P2, or P3 hydrogels (2D culture, *n* = 6), or following suspension of the cells into hydrogels (3D culture, *n* = 3). Laminin (Lam in **C**) and Matrigel (Matr in D) are used as control substrates. Histograms in (**E**,**F**) show the proportion of TUNEL-positive Schwann cells in hydrogels (*n* = 3) and production of growth factor VEGF (*n* = 3) in 3D culture. Error bars show the S.E.M., nd, not detected. *p*-values are indicated as follows: * *p* ≤ 0.05, ** *p* ≤ 0.01 and *** *p* ≤ 0.001; ns, not significantly different. Scale bar, 25 µm.

Pepsin digestion of decellularized nerve tissue resulted in a homogenous solution (‘‘liquid hydrogel’’) which solidified at 37 °C when the tissue weights were in the range of 8 mg/mL to 16 mg/mL. Macroscopically, all hydrogels had a cloudy appearance. Only partial solidification occurred at a concentration of 4 mg/mL. The gels prepared at a concentration of 8 mg/mL deformed easily when lifted with a spatula. Both 12 mg/mL and 16 mg/mL hydrogels were very soft, but could be lifted with a small spatula without losing their integrity (Figure 4A, gels 12 mg/mL). Using absorption measurements, we observed that all hydrogels started to solidify after 5 min at 37 °C, and did not change significantly after 15 min (Figure 4B). For subsequent in vitro and in vivo experiments, we used hydrogels at 12 mg/mL.

A protein array of various cytokines and chemokines in the hydrogels demonstrated that the P2 hydrogel expressed the highest number of the molecules with known effects on nerve regeneration (Supplementary Figure S1).

Next, we assessed the effects of the hydrogels on Schwann cell proliferation, viability, and production of growth factors. All hydrogels supported cell proliferation when the Schwann cells were seeded on top of the gels (2D culture). The rate of cell proliferation on P1 hydrogels was lower than on P2 and P3 hydrogels and laminin coated surfaces (Figure 4C). When Schwann cells were suspended in the hydrogels (3D culture), P1 and P2 hydrogels supported cell viability, but no metabolic activity was detected in the P3 hydrogels (Figure 4D). Therefore, we subsequently determined the number of apoptotic cells using TUNEL staining. The P3 hydrogel induced significant cell death as early as one day in culture, and nearly all cells were TUNEL-positive after the 5th day in culture (Figure 4E). P1 and P2 hydrogels showed lower levels of apoptotic cells (Figure 4E), consistent with the maintenance of viable, non-proliferative cells indicated by the results of the Alamar Blue assay (Figure 4D). Secretion of vascular endothelial growth factor (VEGF) by Schwann cells was observed only in P1 and P2 hydrogels (Figure 4F). BDNF and NGF were not detected above the sensitivity threshold for the ELISAs (data not shown).

**Figure 5:**
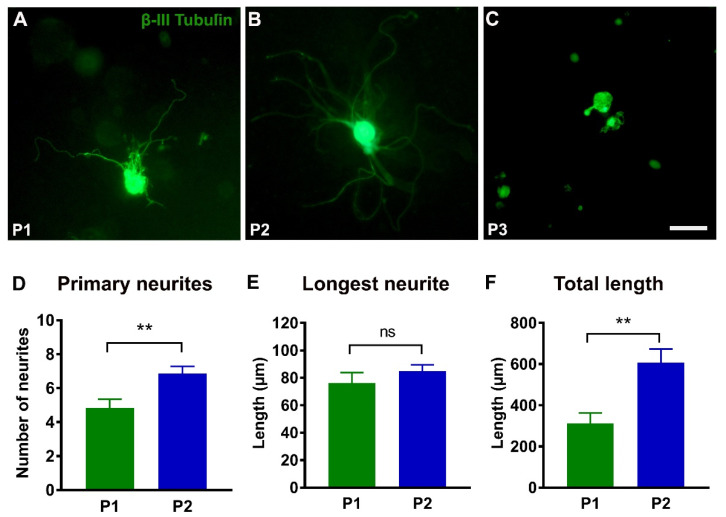
**Neurite outgrowth from dorsal root ganglion (DRG) neurons.** (**A**–**C**): Representative images of the DRG neurons stained for βIII tubulin after 76 h in the P1, P2, and P3 hydrogels. Note that neurons in P3 hydrogel have no neurite outgrowth. (**D**–**F**): Histograms showing the number of primary neurites (P1, *n* = 26 and P2, *n* = 48), the length of the longest neurite (P1, *n* = 26 and P2, *n* = 48), and the total length (*n* = 10) of all neurites in P1 and P2 hydrogels. Error bars show the S.E.M. *p* < 0.01 is indicated by **. ns, not significantly different. Scale bar, 50 µm.

In vitro DRG neurite outgrowth was measured in 3D hydrogel cultures (Figure 5). The P1 and P2 hydrogels permitted significant elongation and arborisation of neurites. Quantitative analyses showed that the P2 hydrogel was more efficient in supporting outgrowth of primary neurites from the cell body and increasing the total neurite length than P1 hydrogel (Figure 5A,B,D,F). The vast majority of DRG neurons in the P3 hydrogel did not form neurites (Figure 5C).

### 2.3. Effects of Hydrogels on Axon Regeneration after Peripheral Nerve Injury

**Figure 6:**
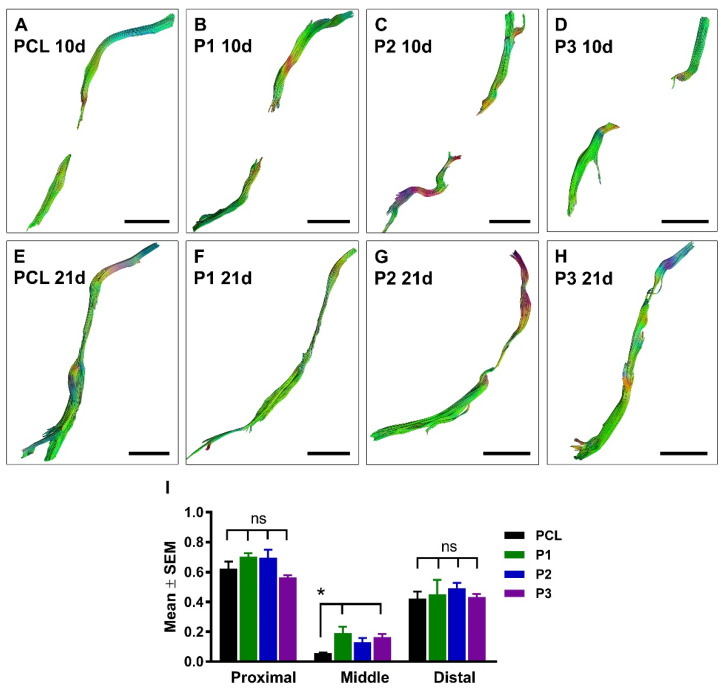
**Diffusion tensor imaging of regenerating axons in the conduits.** Representative axial DTI images at 10 days (**A**–**D**) and 21 days (**E**–**H**) after sciatic nerve injury and repair with empty PCL conduit or PCL conduit filled with P1, P2, or P3 hydrogel. Histogram in (**I**) shows quantification of the fractional anisotropy measured in proximal, middle, and distal ROIs at 21 days after nerve injury and repair (PCL, *n* = 5, P1, *n* = 5, P2, *n* = 7, and P3, *n* = 7). *p* < 0.05 is indicated by * (PCL versus P1 and P3 hydrogels); ns, not significantly different. Scale bar, 5 mm.

PCL conduits alone, or filled with P1, P2, or P3 hydrogels were used to bridge a 10 mm-long gap in rat sciatic nerves. First, we used DTI to evaluate the efficacy of regeneration at 10 days and 21 days after nerve injury and repair (Figure 6A–H and Supplementary Figure S2).

At 10 days after nerve repair, the quantitative anisotropy (QA) maps showed empty conduits in all animals with the exception of one of the P1 scans and one of the P3 scans, where a change in signal gave the impression of intra-conduit fibrous tissue (Supplementary Figure S2). At 21 days postoperatively, a thin fascicular structure reaching the distal nerve stump was detected in all animals except for three rats in the empty PCL group and one animal in the P1 group (Supplementary Figure S2). When performing tractography, no tracts were seen in the 10-day scans except for in one animal in each of the P1 and P3 groups, similar to what was seen in the QA maps. At 21 days, tracts were drawn in all scans except for the three animals in the PCL-alone group and one in the P1 group (Figure 6) as described above for the QA maps.

Overall, the results demonstrated a significant difference between the fractional anisotropy (FA) in the proximal stump and the ROI placed in the middle of the conduit and at the distal stump of the conduit at both 10 and 21 days postoperatively (Figure 6I). We found a lower FA value in the middle of the conduit of the PCL group when compared with P1 and P3 hydrogels in the 21-day scans, possibly suggesting fewer or less densely packed axons passing through the PCL conduit without hydrogel.

**Figure 7:**
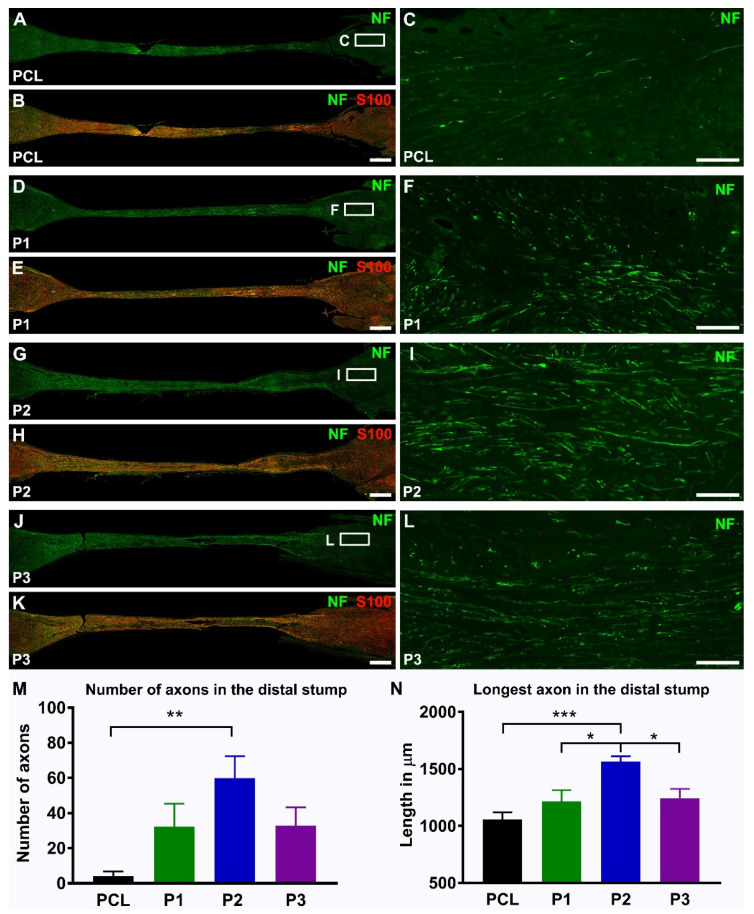
**The effects of hydrogels implantation on regeneration into the distal nerve stump.** Representative longitudinal sections demonstrate neurofilament-labelled axons alone (NF in **A**,**D**,**G**,**J**) or in combination with S100-stained Schwann cells (S100 in **B**,**E**,**H**,**K**)) in the empty PCL conduit (PCL) and in PCL conduits filled with hydrogels (P1, P2, and P3) at 3 weeks following nerve injury and repair. Boxed areas in (**A**,**D**,**G**,**J**) are enlarged in (**C**,**F**,**I**,**L**). Note that regenerating axons reached the distal nerve stump in all experimental groups. Histograms in (**M**,**N**) show the number of regeneration axons and the length of the longest axon in the distal nerve stump (PCL, *n* = 5, P1, *n* = 5, P2, *n* = 7, and P3, *n* = 7). Error bars represent the S.E.M. *p*-values are indicated as follows: * *p* ≤ 0.05, ** *p* ≤ 0.01, and *** *p* ≤ 0.001. Scale bars: 750 µm in the images with conduit reconstructions and 100 µm in the insertions from boxed areas.

Animals were sacrificed at 22 days post-surgical repair, and the conduits and attached proximal and distal nerve stumps were analysed morphologically using immunostaining for neuronal and glial markers (Figure 7). In agreement with the DTI findings, no regenerating axons were detected in the distal nerve stump in the corresponding animals with empty PCL tubes, and only single axons were observed reaching the distal nerve stump in one animal from the P1 experimental group. However, quantitative analysis of the number of regenerating fibres did not reveal any significant differences in the middle of the conduit, but demonstrated differences in the proximal and distal stumps (Figure 7 and Supplementary Figure S3). Reinnervation of the distal nerve stump is the most important outcome in short-term animal experiments, and our results showed that the P2 hydrogel supported a higher number of axons and longer distance of axon regeneration when compared with other hydrogels and empty PCL tubes (Figure 7M,N). However, in contrast to the densely packed fibres inside the conduits, the regenerating axons in the distal nerve stump were spread diffusely.

## 3. Discussion

This study shows that the selection of detergents for nerve tissue decellularization, as well as their concentration and the exposure time, can significantly affect the properties of the resulting hydrogels. We demonstrated that hydrogels P1 and P2 prepared using ionic detergents sodium dodecyl sulfate (SDS) and sodium deoxycholate (SDC), respectively, supported Schwann cell proliferation and secretion of VEGF, and allowed neurite outgrowth from the sensory DRG neurons in 3D hydrogel cultures. P2 hydrogel was the most effective in promoting axon regeneration into the distal nerve stump following sciatic nerve injury and repair.

Decellularization of any tissue always aims at complete removal of the cellular elements with maximal possible preservation of the ECM. However, the type and the concentration of detergent used for decellularization can affect the biochemical and structural composition as well as the ultrastructure of the ECM [18]. The increased concentration of the detergents during decellularization of the urinary bladder results in an increase of detergent remnants in the tissue, which affects cell migration during recellularization [29]. Our results demonstrate that a combination of ionic and non-ionic detergents can reduce the concentration and the exposure time.

The selection of the detergents for this study was based on the tissue properties and previously reported decellularization protocols, including protocols for peripheral nerve tissue [30,31]. Although SDS and SDC are the most common ionic detergents used for decellularization [31], we also tested the efficacy of the organic solvent tri-n-butyl phosphate (TnBP), which is known to dissolve lipids while minimally affecting proteins [32]. Nerve tissue is rich in lipids, mainly in a form of myelin produced by Schwann cells, and a recent study shows that TnBP is more effective than SDS for decellularization of the rat sciatic nerve [33]. Our data suggested that TnBP did not efficiently remove growth-inhibitory MAG [34] from the nerve tissue, and the resulting P3 hydrogels induced apoptosis of the Schwann cells in 3D cultures. Removal of MAG was not observed using P1 and P2 protocols. In our preliminary experiments, in which ethylene diamine tetra acetic acid (EDTA) was added to detergent solutions for disruption of cell attachment to ECM [18], the MAG removal was effective (data not shown). However, the resulting liquid hydrogels did not solidify, which might be attributable to the inhibited collagen fibrillogenesis [35,36].

We hypothesized that another approach to minimize the negative effects on the ECM from using high concentration ionic detergents SDS and SDC [37,38] is to combine them with non-ionic detergent, which is less effective in removing cellular components but, at the same time, could provide better preservation of the matrix proteins. We found evidence that a combination of non-ionic and ionic detergents can perform better than each compound alone, by lowering the critical micelle concentration (concentration at which detergent molecules form a micelle that will disrupt the cells from ECM) and reducing the surface tension [39,40]. A recent study investigating decellularization of the pancreas has successfully applied the same approach of combining detergents [41]. In our study, we used non-ionic surfactant and Triton™ X-100 emulsifier in combination with ionic detergents SDS and SDC. However, in the protocol P3, we treated nerve tissue with Triton™ X-100 and TnBP separately because their mixture resulted in a cloudy and milky solution.

Previous studies investigating nerve decellularization mainly used 3% Triton™ X-100 and 4% SDC for up to 72 h [42,43,44,45,46,47,48,49]. By following our hypothesis of combining detergents, we could reduce these concentrations to 1% for Triton™ X-100 and 2% for SDC. The concentrations of detergents could be significantly lower than in our study, but then additional enzymatic digestion of the nerve tissue with trypsin is needed, which could affect the structural integrity of the ECM [31,50]. However, at present, there is no consensus on the optimal concentrations of the ionic and non-ionic detergent for maximal preservation of the ECM.

In our experiments, we were able to efficiently remove cellular components in the treated nerves. However, DNA analysis revealed that the SDC-based P2 protocol is superior to the other two protocols, as genomic DNA or DNA with a size larger than 200 bp was not detected following agarose gel electrophoresis. Quantitative analysis of the P2 decellularized nerves showed that the amount of DNA is close to the 50 ng/mg dry weight level, which is considered a standard for a decellularized tissue to be non-immunogenic [51]. Thus, our observations corroborate previous reports that SDC is more efficient than SDS for nerve decellularization [52].

There are also different reports on the amount of collagen following nerve decellularization. Cornelison et al. [53] showed an increase in the amount of collagen per tissue mass, whereas our data for collagen quantification in all tested nerves are in line with the results reported by Meder et al. and Lin et al. that showed no difference when comparing native and decellularized nerves [46,54]. In contrast, protocol P2 with SDC resulted in significantly reduced levels of sulfated glycosaminoglycans (GAG). Chondroitin sulfate proteoglycans with GAG side chains are known inhibitors of axonal regeneration in the adult nervous system [55]. Hudson’s protocol for nerve decellularization shows that additional digestion of GAG with the enzyme chondroitinase ABC during nerve decellularization can improve regenerative effects [56]. Our results also demonstrate that P2 hydrogel had the lowest levels of GAG, and was the most effective in stimulating neurite outgrowth in vitro and following sciatic nerve injury and repair in animal models.

Though preparation of hydrogels from decellularized tissue by enzymatic digestion is a common method [57], it has not always been successful [58,59]. The reasons could be the type of the tissue, ECM composition, and protein structure, as well as the type of detergent and treatment conditions [60,61]. In this study, we did not observe any negative effects of decellularization on hydrogel solidification, and the selected 12 mg/mL hydrogel concentration is comparable to the 9–13 mg/mL concentration widely used in other studies [46,53]. The higher concentrations of hydrogels could negatively affect cell viability and tissue remodelling upon transplantation [54,62].

Proteomics analysis of the nerve-derived hydrogels in previous reports showed preservation of several structural and functional proteins including collagens, laminins, and fibronectin, with known important roles in nerve regeneration [44,63]. Recently, it has been shown that nerve-derived hydrogels could contain neurotrophic factors [54]. Our data additionally demonstrate the presence of various cytokines and chemokines with known effects on axon regeneration, such as FGF-21 (fibroblast growth factor), IGF-2 (Insulin growth factor), ANG-1 (Angiopoietin), EPO (Erythropoietin), and IL-4 (Interleukin).

The ECM and growth-promoting factors in nerve-derived hydrogels are most likely responsible for the reported proliferation of Schwann cells and neurite outgrowth in previous studies using 2D culture system [42,44,45,46,63]. Our findings with 2D culture systems supported these observations, and demonstrated that all tested hydrogels can stimulate Schwann cell proliferation. However, when cells were suspended in hydrogels for 3D culture models, only P1 and P2 hydrogels supported cell viability, secretion of growth factor VEGF, and neurite outgrowth from sensory DRG neurons.

In accordance with previous observations [42,46,47,53], the present study showed that hydrogels can support axon regeneration across the 10 mm-long nerve gap in the injured sciatic nerve of adult rat. The regeneration was assessed using diffusion tensor imaging (DTI) and immunostaining for neuronal and glial markers.

DTI is a non-invasive MRI-based technique that can assess pathological changes in both the central and peripheral nervous systems at multiple times points, without the need to sacrifice experimental animals. As the tissue architecture of peripheral nerves consists of parallel-aligned axons enveloped in connective tissue, the diffusion of water molecules occurs primarily along the axis of the nerve, and upon injury, axonal death, or demyelination, the fraction of water molecules diffusing perpendicular to the direction of the nerve increases [64]. This preferred directionality of water molecules is described as the fractional anisotropy (FA), and previous studies have shown that injured peripheral nerves show a decrease in FA compared with healthy nerves [65].

Similar to our earlier observations [65], we found typical for anterograde Wallerian degeneration a decrease in the fractional anisotropy (FA) of the distal nerve stump. The conduits were clearly visualised in the diffusion-weighted images used to reconstruct the quantitative anisotropy maps, and in the 21-day scans a visible fascicle could be seen crossing the gap between the proximal and distal ends of the transected and repaired sciatic nerve. This supports the hypothesis that diffusion-based MRI techniques can be employed to follow regenerating peripheral nerves, also in synthetic conduit materials. The FA in the middle of the conduit increased in all experimental groups at the 21-day scans, compared to the 10-day scan, with the exception of the PCL group. The discrepancy between the FA values and the quantification of axons in histological preparations could be due to a less directed axon regrowth across the gap, sprouting, or diffusion properties of the hydrogels compared with an empty conduit. No statistically significant difference could be detected in the distal end when comparing the 10-day and 21-day scans, suggesting that regenerating axons in the distal nerve stump at 21 days postoperatively do not have fascicular organization in the normal peripheral nerve, or are too few to affect the diffusion parameters at the area of interest at this early time point. This observation was supported by morphological analysis showing a diffuse pattern of growth of small number of axons in the distal stump.

## 4. Materials and Methods

### 4.1. Ethical Statement

Isolation of the Schwann cells and dorsal root ganglion neurons for cell culture and implantation experiments was performed on adult female Sprague-Dawley rats (12–16 weeks old, *n* = 30; Charles River Laboratories, Sulzfeld, Germany). The animal care and experimental procedures were carried out in accordance with Directive 2010/63/EU of the European Parliament and of the Council on the Protection of Animals used for Scientific Purposes, and were approved by the Animal Review Board at the Court of Appeal of Northern Norrland in Umeå (ethical permits DNR #A15-20 and #A31-19). Porcine vagus nerves (*n* = 20) were harvested from the discarded samples of the pig’s internal organs used for research and educational purposes at Umeå University (ethical permit DNR 6.7.18-5352/17).

### 4.2. Decellularization of Nerve Tissue

Approximately 30 cm-long pieces of porcine vagus nerves were collected in cold PBS. The nerves were cleaned of blood, fat, and surrounding connective tissue, frozen, and stored at −85 °C before processing. The nerves were thawed at room temperature, cut into 10 cm-long pieces, washed overnight in distilled water (DH_2_O), and randomly assigned to one of the tested protocols. All steps in the decellularization protocols involved agitation at 200 rpm and were performed at room temperature. Three different protocols were used for nerve decellularization (Figure 1). In the first protocol (P1), the nerves were incubated for 6 h in a solution containing 2% of non-ionic surfactant, Triton™ X-100 (T8787, Sigma-Aldrich Sweden AB, Stockholm, Sweden) emulsifier, and 0.2% of an ionic detergent sodium dodecyl sulfate (SDS, L3771, Sigma-Aldrich Sweden AB, Stockholm, Sweden). In the second protocol (P2), the nerves were treated for 6 h in a solution of 1% Triton™ X-100 and 2% of an ionic detergent sodium deoxycholate (SDC, S1827, Sigma-Aldrich Sweden AB, Stockholm, Sweden). In the third protocol (P3), the nerves were first incubated in 3% solution of Triton™ X-100 for 3.5 h, washed briefly in distilled water, and then agitated for an additional 3.5 h in 3% solution of the organic solvent tri-n-butyl phosphate (TnBP, A16084, Alfa Aesar by Thermo Fisher GmbH, Kandel, Germany). All detergent solutions were supplemented with 10 kIU/mL aprotinin (A3428, Sigma-Aldrich Sweden AB, Stockholm, Sweden). The nerves were washed in DH_2_O overnight, and in total the treatment with Triton™ X-100 and detergent was repeated 4 times in P1 and P2 protocols and 6 times in P3 protocol. Finally, the nerves were treated with 2 U/mL Benzonase^®^ endonuclease (1.01654.0001, Merck Life Science AB, Solna, Sweden) in hypertonic buffer (50 mM Tris-HCl, 1 mM MgCl_2_, pH 7.5) for 4 h at 37 °C and washed in DH_2_O for 48 h.

### 4.3. DNA Quantification

Untreated and decellularized pieces of nerves (*n* = 5 per group) were lyophilized, incubated in RLT lysis buffer from a DNA isolation kit (69504, QIAGEN AB, Kista, Sweden) for 1 h, and DNA was isolated and quantified using a NanoDrop™ One spectrophotometer (ND2000, Thermo Fisher Scientific, Stockholm, Sweden). The data were normalized to the tissue weight. For visualization, 10µg DNA from each sample was run on a 1% agarose gel containing 0.00005% ethidium bromide.

### 4.4. Quantification of Collagen and Glycosaminoglycans

Insoluble fibrillary collagen and sulfated glycosaminoglycans (GAG) were quantified in lyophilized normal and decellularized nerves (*n* = 5 per group) using Sircol (S2000, Biocolor Ltd., Carrickfergus, UK) and blyscan (B1000, Biocolor Ltd., Carrickfergus, UK) assay kits, respectively. For the collagen assay, nerves were digested in fragmentation reagent, and for the GAG assay, nerves were digested in papain extraction reagent at 65 °C for 2–3 h. Isolated collagen and GAGs were precipitated with Sircol and blyscan, respectively, centrifuged and released into dissociation reagent. The absorbances at 556 nm and 656 nm, respectively, were measured with a microplate reader (Synergy, BioTek, Santa Clara, CA, USA) and the data was normalized to dry tissue weight and displayed as mg of protein per mg of tissue.

### 4.5. Preparation of Hydrogels and Gelation Kinetics

The decellularized nerves were cut into small pieces, lyophilized, and stored at −20 °C before use. The dry tissue was digested at 20 mg/mL in pepsin solution (1 mg/mL in 0.01 N HCl) until no visible nerve tissue pieces were present (55–60 h). After digestion was complete, the pH was adjusted to 7.4 with 1 N NaOH, and the salt concentration was adjusted to 1× with 20× PBS. The resulting liquid hydrogel was stored at −85 °C before processing. The gelation kinetics for liquid hydrogels were measured for the following concentrations: 4 mg/mL, 8 mg/mL, 12 mg/mL, and 16 mg/mL. The hydrogels were diluted in ice-cold PBS, and 90µL of the P1, P2, or P3 hydrogels (*n* = 5 per group) was added into the 96-well plate. Absorbance at 405 nm was recorded every 5 min during the first 40 min, and then at 60 and 90 min.

### 4.6. Total Protein Isolation and Quantification

The nerve tissue and hydrogels were mechanically homogenized (TissueRuptor, QIAGEN AB, Kista, Sweden) and digested in RIPA lysis buffer (89901, Thermo Fisher Scientific, Stockholm, Sweden) containing protease inhibitor cocktail (P8340, Sigma-Aldrich Sweden AB, Stockholm, Sweden) for 3 min. The digest was centrifuged and the amount of protein was quantified using a Bio-Rad DC protein assay kit (5000111, Bio-Rad Laboratories AB, Solna, Sweden).

### 4.7. Analysis of Cytokines and Chemokines in the Hydrogel

The cytokines and chemokines present in the hydrogels were quantified using the Porcine Cytokine Array C1 (AAP-CYT-1-2, RayBiotech Life, Inc., Peachtree Corners, GA, USA). Briefly, 240 µg protein from native nerve, and P1, P2, and P3 hydrogels were added onto membranes coated with antibodies followed by detection antibodies and HRP-conjugated streptavidin. The signal was detected using chemiluminescence imaging with an Odyssey Fc system (Li-COR Biotechnology GmbH, Bad Homburg, Germany). The intensity of dots was measured with image studio software (Li-COR Biotechnology GmbH, Bad Homburg, Germany). The protein differences between gels were expressed as fold change by normalising average intensity for each protein with the intensity in native nerve.

### 4.8. Schwann Cell Culture

Schwann cells were prepared from the sciatic nerves of adult female Sprague–Dawley rats (*n* = 3), as described by us previously [66]. In brief, the nerves were cut into 1 mm-long pieces and incubated in low glucose DMEM with 10% heat-inactivated fetal calf serum (FCS) and 1% penicillin/streptomycin at 37 °C, 95% humidity, and 5% CO_2_. After 2 weeks, the nerves were enzymatically dissociated with 250U collagenase-I (CLS-I, Worthington Biochemical Corp., Lakewood, NJ, USA) and 1.6U dispase-I (D4818, Sigma-Aldrich Sweden AB, Stockholm, Sweden) for 2 h, triturated, filtered through a 70 µm filter, and centrifuged to pellet the cells. The cells were resuspended in DMEM with 10% FCS, 5 µM forskolin (F6886, Sigma-Aldrich Sweden AB, Stockholm, Sweden), and 50 ng/mL neuregulin (396-HB, R&D Systems, Inc., Minneapolis, MN, USA). After cells became confluent, contaminating fibroblasts were removed by labelling with mouse anti-rat Thy1.1 antibody (1:1000, MAB1406, Millipore by Sigma-Aldrich Sweden AB, Stockholm, Sweden) and complement-mediated lysis (Low Tox M Rabbit Complement, CL3051, Cedarlane®, Burlington, Canada). The purity of Schwann cells was assessed using immunostaining for S-100 protein, and was approximately 90%.

### 4.9. Cell Proliferation Assay on Hydrogels

The AlamarBlue™ assay (BUFO12A, Bio-Rad Laboratories AB, Solna, Sweden) was used to evaluate the effects of hydrogels on Schwann cell attachment and proliferation. For 2D cultures, 48-well plates were coated with 2 µg of hydrogel or laminin (0.5 µg/cm^2^, L2020, Sigma-Aldrich Sweden AB, Stockholm, Sweden) and incubated at 37 °C. After 12 h, Schwann cells were added to the coated wells (6000 cells per well, *n* = 6 wells/hydrogel). To study Schwann cells proliferation in 3D cultures, liquid hydrogels (12 mg/mL) containing Schwann cells (5.5 × 10^6^ cells/mL) were prepared and 50 µL of each hydrogel was added per well (*n* = 3 wells (hydrogel). Matrigel (8–12 mg/mL, E1270, Sigma-Aldrich Sweden AB, Stockholm, Sweden) was used as control for 3D culture. At 24 h, 72 h, and 120 h, Schwann cell growth medium containing 10% AlamarBlue™ was added, incubated for 8 h, and the optical density of AlamarBlue™ was measured using a microplate reader (Synergy, Biotek, Santa Clara, CA, USA) at 570 nm with background correction at 600 nm.

### 4.10. Enzyme Linked Immunosorbent Assay (ELISA)

ELISA was used to study secretion of growth factors from the Schwann cells suspended in the tested hydrogels. Schwann cells were added to the liquid hydrogels (5.5 × 10^6^ cells per 1 mL of 12 mg/mL hydrogels), and 50 µL of the mixture was added per well in a 24-well plate (*n* = 3 per hydrogel type). After solidification, 300 µL of Schwann cell growth medium was added to the wells. The medium was changed every 24 h. The medium was collected at 24, 72, and 120 h, pooled for each hydrogel type, and growth factors were analysed using rat VEGF-A ELISA kit (ELR-VEGF-1, RayBiotech Life, Inc., Peachtree Corners, GA, USA), rat BDNF ELISA kit (ELR-BDNF-1, Ray Biotech Life, Inc., Peachtree Corners, GA, USA), and NGF beta rat ELISA kit (ERNGF, Invitrogen by by Thermo Fisher Scientific, Stockholm, Sweden).

### 4.11. Neurite Outgrowth Assay in Hydrogels

Cultures of dorsal root ganglia (DRG) neurons were prepared from adult rats (*n* = 3) as described previously [67]. In brief, DRGs were collected in cold Neurobasal™-A medium (10888-022, Gibco by Thermo Fisher Scientific, Stockholm, Sweden), digested in 250 µg collagenase-IV (C5138, Sigma-Aldrich Sweden AB, Stockholm, Sweden ) for 1 h 45 min and in 2.5% trypsin (T2605, Sigma-Aldrich Sweden AB, Stockholm, Sweden) for 30 min. After neutralizing with FCS and washing in neurobasal medium, DRG were mechanically dissociated, added onto 15% BSA, and centrifuged. Pelleted neurons were resuspended in liquid hydrogels (44,500 neurons per ml of 12 mg/mL hydrogels), and 30 µL of the mixture was added per well in a 24-well plate (*n* = 3 per hydrogel type). After solidification, the hydrogels with neurons were incubated for 76 h in Neurobasal™-A medium supplemented with B-27 (17504044, Gibco by Thermo Fisher Scientific, Stockholm, Sweden) and 1% L-glutamine–penicillin/streptomycin solution (G6784, Sigma-Aldrich Sweden AB, Stockholm, Sweden).

### 4.12. Preparation of the Tubular Conduits with Hydrogels

Tubular conduits were made from non-toxic biodegradable polycaprolactone (PCL, average Mn 80,000; 440744, Sigma-Aldrich Sweden AB, Stockholm, Sweden). PCL pellets were heated to 80 °C and then moulded around a steel rod (2 mm O.D.), re-heated, and stretched to produce a thin tube. The tube had an average wall thickness of 144 ± 18 µm, and was cut into 14 mm-long pieces. The conduits were treated with 10 N NaOH for 1 h at room temperature to increase the pit patterning of the surface and to improve cell attachment [68]. After washing, they were sterilized under UV light for 30 min. Liquid hydrogels P1, P2, or P3 (12 mg/mL, 32 µL) were filled into the tubes and allowed to solidify at 37 °C for 30 min. DMEM was added, and conduits were kept overnight in an incubator and used for implantation the next day.

### 4.13. Sciatic Nerve Injury Model

All surgical procedures were performed under 2% isoflurane anaesthesia in combination with buprenorphine (Temgesic, Indivior Europe Ltd., Dublin, Ireland; 0.025 mg/kg, subcutaneous). The postoperative treatment included the analgesic, Finadyne (Schering-Plough, Denmark; 2.5 mg/kg, s.c.), normal saline (2 mL, s.c.), and benzylpenicillin (Boehringer Ingelheim; 60 mg, s.c.). Each animal was housed alone in a cage after surgery and exposed to 12-h light/dark cycles, with free access to food and water. The sciatic nerve was exposed on the left leg under an operating microscope (Carl Zeiss, Jena, Germany) and divided about 5 mm distal to the exit point from the sciatic notch [65,67,69]. The animals were randomly divided into four experimental groups: (i) empty PCL conduit (*n* = 5), (ii) PCL conduit filled with P1 hydrogel (*n* = 5), (iii) PCL conduit filled with P2 hydrogel (*n* = 7), and (iv) PCL conduit filled with P3 hydrogel (*n* = 7). The 14 mm-long conduit was placed into the gap and 2 mm of the proximal and distal ends of the divided sciatic nerve were introduced into the conduit and secured with 10.0 Ethilon (Ethicon) sutures, creating a gap of 10 mm between the proximal and distal ends. The wound was then closed in layers, and the rats were returned to their cages with heating pads for recovery.

### 4.14. Diffusion Tensor Imaging (DTI) and Data Analysis

At 10 and 21 days post-operatively, the animals were scanned using a 9.4 T Bruker BioSpec 94/20 USR system. Previous results from our laboratory have demonstrated that regenerating axons do not cross the 10 mm-long nerve injury gap at 2 weeks, but reach the distal stump after 3–4 weeks postoperatively [67,69,70]. Therefore, we used 10 days and 21 days for DTI imaging to evaluate the sensitivity of this technique to differentiate between the non-regenerating and regenerating axons in the conduit. For histological analysis, the animals were sacrificed on the day after DTI experiments, i.e., 22 days after nerve injury and repair.

The general setup has been described previously [65]. The scanner was connected to a rat brain array coil combined with an 87 mm QUAD resonator coil, and running ParaVison^®^ 6.1 software (Bruker Biospin Group, Bruker Corporations, Munich, Germany). The animals were kept under 2% isoflurane anaesthesia with respiration monitoring by use of a respiration pillow (SA Instruments Inc., Stony Brook, USA) throughout the scans. Data were collected from 4 separate DTI EPI Spin Echo diffusion scans. A multi-shell diffusion scheme was used with b-values 1000, 1500, 2000, and 2500 s/mm^2^, and a total of 32, 32, 64, and 64 directions respectively. Echo time (TE) was 0.4608 ms, and repetition time (TR) was 2000 ms. The diffusion time was 10.5 ms, and the diffusion encoding duration 4.5 ms. Isometric voxels of 0.4 mm were obtained. The diffusion data was reconstructed and analysed in DSI Studio (13 December 2018 Build) using generalized q-sampling imaging (GQI) [71] with a diffusion sampling length ratio of 0.6. GQI is a model-free approach with outputs comparable to complex q-space methods, and produces quantitative anisotropy (QA) maps with better contrast than traditional fractional anisotropy (FA) maps when identifying peripheral nerves [72]. It produces the traditional diffusion parameters FA, mean diffusivity (MD), axial diffusivity (AD), and radial diffusivity (RD). Regions of interest (ROIs) of 0.5 mm^3^ were placed in three locations—at the proximal stump entering the conduit, in the middle part of the conduit, and in the distal stump at the end of the conduit. The FA values at the three locations were extracted from the ROIs. To detect any fibres passing through the conduit, the ROI in the middle of the conduit was used as a seed ROI for tractography. The tractography visualisation was performed by tracking all fibres passing through each of the ROIs. Termination index was a normalized quantitative anisotropy (nQA), with the threshold set to random. A sub-voxel step size of 0.2 mm was used, with an angular threshold of 40 degrees. Tracts shorter than 8 mm were removed from the analysis.

### 4.15. Tissue Processing

At 22 days postoperatively, the animals were sacrificed with an overdose of sodium pentobarbital (240 mg/kg, i.p.). The conduits were removed, fixed in 4% paraformaldehyde (PFA) in 0.1 M phosphate buffer (pH 7.4) overnight at 4 °C, cryoprotected in 10% and 20% sucrose for 2–3 days, and frozen in liquid isopentane. Serial longitudinal 12 µm-thick sections were cut on a cryomicrotome (Leica Microsystems GmbH, Wetzlar Germany), thaw-mounted in pairs onto SuperFrost^®^ Plus slides, dried at 37 °C for 1 h, and stored at −20 °C before processing.

### 4.16. Histological Staining and Immunohistochemistry

All decellularized nerves and hydrogels for in vitro studies were prepared as described above, but fixation time in 4% PFA was 30 min. Collagen in the decellularized nerves was stained with Trichrome Stain (Masson) Kit (HT-15, Sigma-Aldrich Sweden AB, Stockholm, Sweden ) in combination with Weigert’s Iron Hematoxylin solution (HT1079, Sigma-Aldrich Sweden AB, Stockholm, Sweden) for cell nuclei. Laminin- and myelin-associated glycoprotein in the decellularized nerves, cultured cells in the hydrogels, and regenerating axons and glial cells in the conduits were labelled using immunochemistry. After blocking with normal serum, the following primary antibodies were used: rabbit anti-laminin (1:500, ab11575, Abcam Cambridge, United Kingdom), goat anti rat MAG/Siglec-4a (1:60, AF538, R&D Systems, Inc., Minneapolis, MN, USA), rabbit anti-S100 protein (1:1000, Z0311, Dako Agilent, Santa Clara, CA, USA), rabbit anti-β-Tubulin III (1:200, T2200, Sigma-Aldrich Sweden AB, Stockholm, Sweden), and mouse anti-neurofilament 200 (NF 200, 1:400, N0142, Sigma-Aldrich Sweden AB, Stockholm, Sweden). All primary antibodies were applied for 2 h at room temperature. After rinsing in PBS, corresponding secondary antibodies Alexa Fluor^®^ 488 and Alexa Fluor^®^ 568 (1:1000; Molecular Probes, Invitrogen by Thermo Fisher Scientific, Stockholm, Sweden) were applied for 1 h at room temperature in the dark. The slides were coverslipped with ProLong mounting media containing DAPI (G3250, Promega Biotech AB, Nacka, Sweden). The staining specificity was tested by omission of primary antibodies. TUNEL staining was performed using DeadEnd^TM^ Fluorometric TUNEL System (G3250, Promega Biotech AB, Nacka, Sweden), following the manufacturer’s instructions.

### 4.17. Image Analysis and Quantification

Images were captured using a Nikon Eclipse E80i microscope coupled to a Nikon DS-U2 digital camera. The outgrowth of neurites from DRG neurons was traced with ImageJ software in P1 hydrogels (*n* = 26 neurons) and P2 hydrogels (*n* = 48 neurons). The total number of primary neurites and the longest neurite outgrowth was determined in all captured neurons. Only neurites longer than the diameter of parent cell body were analysed. The total neurite length was measured in 10 neurons that had longest neurite outgrowth in each hydrogel group. TUNEL-positive and negative Schwann cells were counted manually (9 images per hydrogel, at least 50 cells per frame). The number of regenerating axons was quantified in 4–5 longitudinal sections taken from the middle part of the tissue cable bridging the proximal and distal nerve stumps in the conduit. Axon profiles crossing a line perpendicular to the direction of the conduit were counted at the distance of 100 µm into the proximal stump, 200, 500, and 800 µm from the proximal stump, and at 100 µm into the distal stump. Axon regeneration distance in the distal stump was measured using an optical microgrid.

### 4.18. Statistical Analyses

Statistical analyses were performed using GraphPad Prism version 7.00 for Windows, (GraphPad Software, La Jolla CA, USA, www.graphpad.com (accessed on 20 February 2017), Prism perpetual license to Lev Novikov, accessed on 20 February 2017). Mann–Whitney U test and one-way analysis of variance (ANOVA), followed by Tukey’s multiple comparisons test, were performed to determine all statistically significant differences. Data on all graphs are presented as the mean and the standard error of the mean (SEM). *p*-values are denoted as follows: * *p* ≤ 0.05, ** *p* ≤ 0.01, and *** *p* ≤ 0.001.

## 5. Conclusions

In summary, the results of this study show that peripheral nerve-derived hydrogels alone, without any complementary treatments, can support axon regeneration following peripheral nerve injury. However, a combination of hydrogels with transplanted cells, cell products, or gradient of growth factors, to further increase the regenerative response from the injured neurons, and to stimulate long-distance regeneration, should be considered.

## Data Availability

All datasets generated and/or analysed during the current study are available from the corresponding author upon reasonable request.

## Supplementary Materials

The following supporting information can be downloaded at: https://www.mdpi.com/article/10.3390/ijms23158746/s1.
