# Supplementary material for: Efficacy of Nerve-Derived Hydrogels to Promote Axon Regeneration Is Influenced by the Method of Tissue Decellularization
Source: Int J Mol Sci. 2022 Aug 6;23(15):8746. doi: 10.3390/ijms23158746 (PMC9369339; doi:10.3390/ijms23158746)
Supplement: Supplementary file 1 [file ijms-23-08746-s001.zip › ijms-1827447-supplementary.pdf]

SUPPLEMENTARY MATERIALS

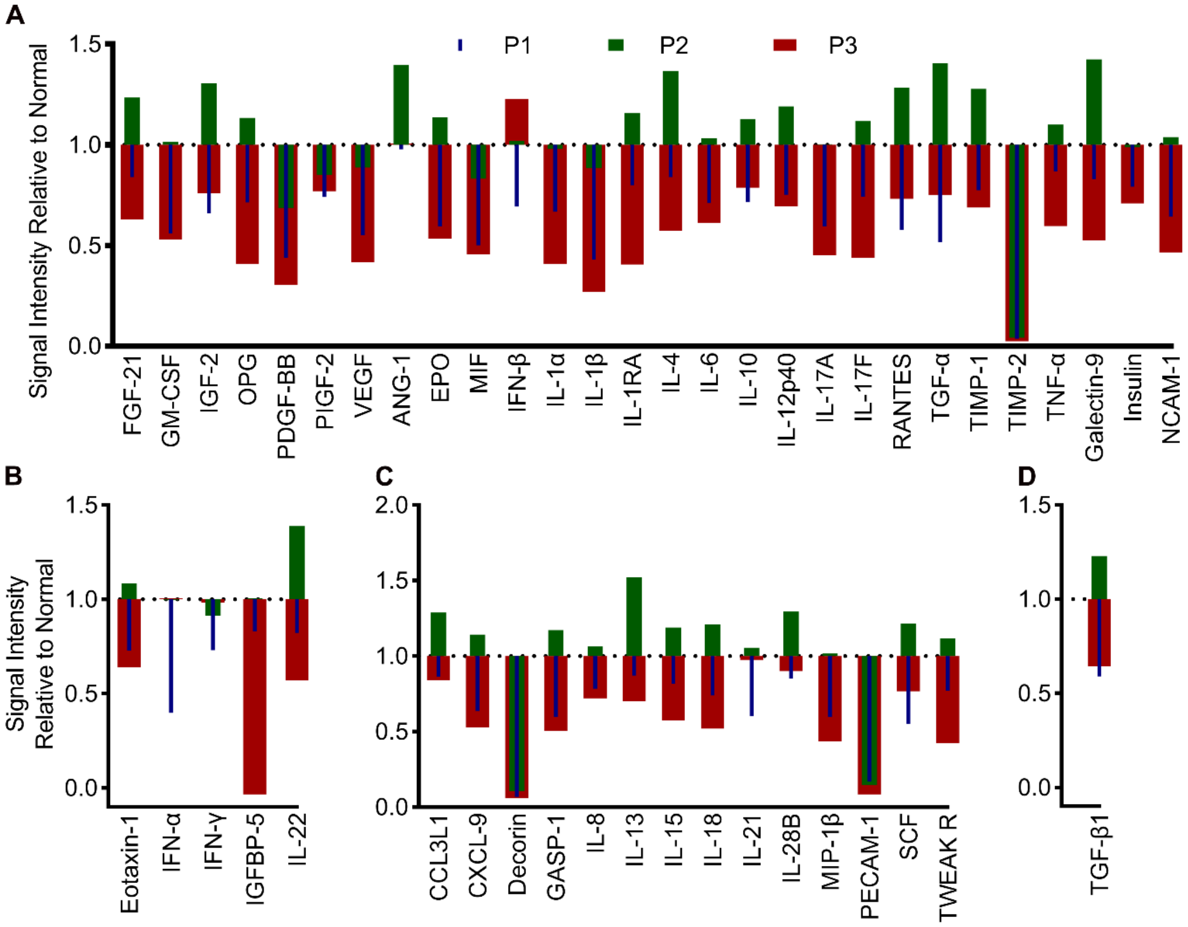

Supplementary Figure S1: Analysis of cytokine/chemokine content in hydrogels. The cytokines and chemokines in the P1, P2 and P3 hydrogels are normalized to the untreated normal nerves and subdivided into four groups: (A) with known positive effects on nerve regeneration, (B) with known negative effect on nerve regeneration (C) cytokines and chemokines with undefined function in nerve regeneration and (D) cytokine with demonstrated both positive and negative effect on nerve regeneration.

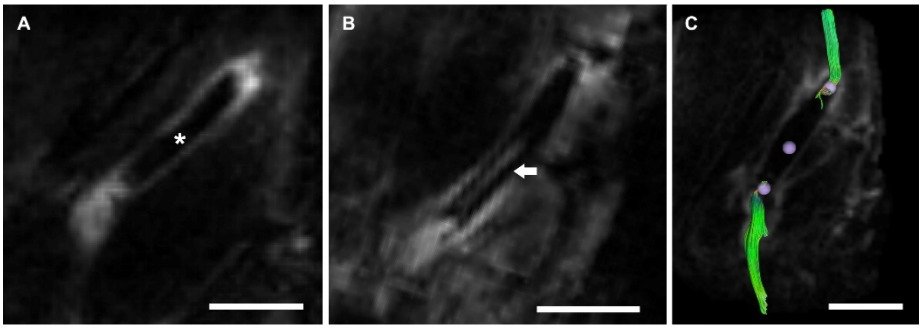

Supplementary Figure S2: Raw data for quantitative DTI analysis of regenerating axons in the conduits. Greyscale QA maps at 10 days (A) and 21 days (b) after sciatic

nerve injury and repair. Asterisk (A) indicates empty PCL conduit. Arrow (B) indicates fascicle passing through the PCL conduit. Image in (C) demonstrates the placement of the ROIs (filled circles) in the proximal nerve stump, in the middle of the conduit and in the distal nerve stump. Nerve stumps in (C, green colour) are traced with DTI. Scale bar, 5 mm.

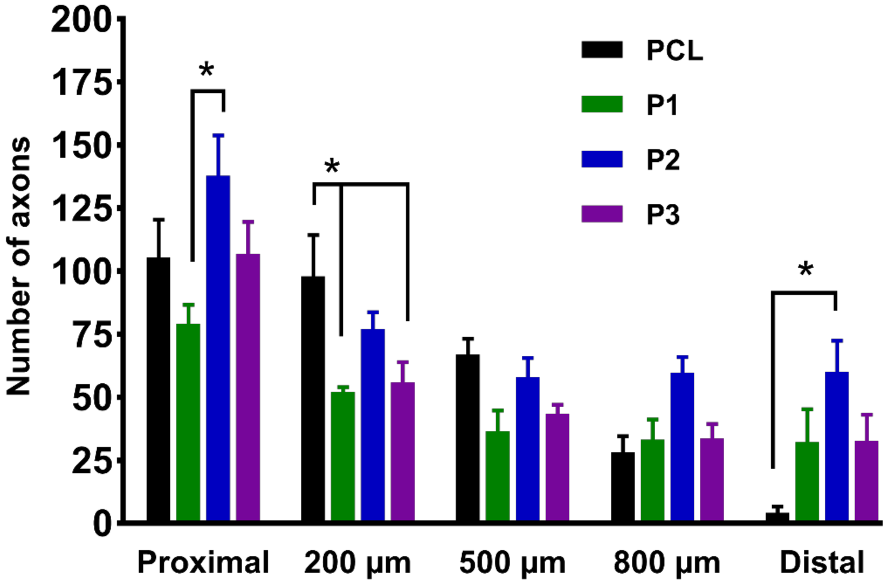

Supplementary Figure S3: Quantitative analysis of regenerating axons in the conduits. Histogram shows the number of regeneration axons in the empty PCL conduit (PCL) and in PCL conduits filled with hydrogels (P1, P2, P3) at different distances from the proximal nerve stump and in the distal nerve stump. Error bars represent the S.E.M. $P<0.05$ is indicated by *.
